# Vitamin D and lipopolysaccharide jointly induce a distinct epigenetic and transcriptional program in human monocytes

**DOI:** 10.1038/s41598-025-10921-2

**Published:** 2025-07-28

**Authors:** Mariusz Jankowski, Emmi Hämäläinen, Mari Taipale, Sami Heikkinen, Carsten Carlberg

**Affiliations:** 1 Institute of Animal Reproduction and Food Research, Polish Academy of Sciences, ul. Trylińskiego 18, Olsztyn, 10-683 Poland; 2 Institute of Biomedicine, University of Eastern Finland, Kuopio, Finland; 3 Present address: Faculty of Medicine and Health Technology, Tampere University, Tampere, Finland; 4 Present address: A. I. Virtanen Institute, University of Eastern Finland, Kuopio, Finland

**Keywords:** Immune challenge, Vitamin D, Lipopolysaccharide, Chromatin accessibility, Transcriptome, Responsive genes, Gene regulation in immune cells, Endocrinology

## Abstract

Pathogen-associated molecular patterns such as lipopolysaccharide (LPS) mimic immune responses triggered by bacterial infections. The hormonally active form of vitamin D_3_, 1α,25-dihydroxyvitamin D_3_ [1,25(OH)_2_D_3_], supports innate immunity, but its molecular mechanisms remain incompletely understood. We investigated epigenomic and transcriptomic changes in THP-1 monocytes that were either unprimed or primed for 24 h with 1,25(OH)_2_D_3_ or LPS, followed by a second 24-hour stimulation with 1,25(OH)_2_D_3_, LPS, or their combination. Epigenome profiling *via* ATAC-seq revealed that co-stimulation with 1,25(OH)_2_D_3_ and LPS induces substantially more chromatin accessibility changes than either treatment alone, with up to 81% of altered regions uniquely responsive to the combination. Motif enrichment analysis highlighted JUN/FOS transcription factors as key regulators of this synergistic response. Transcriptomic analysis *via* RNA-seq mirrored these findings, though fewer genes than chromatin regions were affected. Notably, under 1,25(OH)_2_D_3_-primed conditions, 331 genes exhibited synergistic expression changes upon co-treatment, meaning that their responses significantly deviates from the additive effects of the individual stimulations. This includes 264 genes previously unrecognized as vitamin D targets. Functional annotation revealed that these genes are primarily linked to monocyte and T cell differentiation, in contrast to classical vitamin D targets associated with inflammation. In conclusion, our findings provide mechanistic insight into how vitamin D modulates inflammation through epigenetic and transcriptional reprogramming.

**Supplementary Information:**

The online version contains supplementary material available at 10.1038/s41598-025-10921-2.

## Introduction

Following infection or vaccination, innate immune cells, such as circulating monocytes and tissue-resident macrophages, undergo persistent alterations in their epigenetic landscape, gene expression profiles, and cellular functions^1^. This phenomenon, known as immune cell reprogramming, is often triggered by pathogen-associated molecular patterns, which are structural components predominantly or exclusively present on microbial surfaces^2^. A key example is LPS, a glycolipid located in the outer membrane of Gram-negative bacteria^3^. LPS activates monocytes and macrophages through TLR4 (Toll-like receptor 4), initiating a cascade of intracellular signaling events^4^. This pathway involves kinases from the mitogen-activated protein kinase (MAPK) family, ultimately leading to the activation of key transcription factors such as CREB1 (cAMP response element-binding protein 1), AP1 (activator protein 1), and NFκB (nuclear factor kappa B). Through these mechanisms, LPS acts as a model stimulus for mimicking bacterial infections, eliciting substantial transcriptomic changes in innate immune cells^5,6^.

The biological outcome of this process, referred to as trained immunity, is characterized by an amplified response upon subsequent encounters with microbial antigens. This includes prolonged production of pro-inflammatory cytokines and an enhanced capacity to eradicate pathogens^7,8^. While trained immunity generally provides a protective advantage, it can also contribute to pathological inflammation, particularly in conditions such as sepsis or autoinflammatory diseases^9^.

Vitamin D, classified as a secosteroid, exerts its biological effects primarily through its active metabolite, 1,25(OH)_2_D_3_, which functions as a ligand for the vitamin D receptor (VDR), a transcription factor belonging to the nuclear receptor superfamily^10^. Unlike LPS, which influences gene expression indirectly *via* signaling cascades, 1,25(OH)_2_D_3_ directly regulates gene transcription through VDR activation^11^.

Vitamin D’s overarching role involves supporting the energy balance and survival mechanisms of VDR-expressing cells^12^. Its key physiological functions include regulating calcium homeostasis to promote proper bone mineralization^13^ and modulating immune responses to maintain immune equilibrium^14^. Through its immunomodulatory properties, vitamin D enhances the host’s defense mechanisms against infectious agents^15^ while simultaneously mitigating the risk of immune overactivation, as observed in autoimmune disorders^16^. This dual role contributes to an optimized immune response, benefiting both the innate and adaptive arms of the immune system. Conversely, vitamin D deficiency is frequently linked to a higher incidence of complications from infectious diseases, such as tuberculosis^17^ and COVID-19^18^, as well as chronic inflammatory conditions like inflammatory bowel disease. Furthermore, inadequate vitamin D levels are associated with an increased risk of developing and progressing autoimmune diseases^19,20^including multiple sclerosis.

Both vitamin D, its metabolites, and synthetic analogs possess not only preventive potential against various diseases^21^ but are also employed therapeutically, for example, in treating autoimmune conditions such as psoriasis^22^. In this study, we investigated potential functional interactions between vitamin D and LPS signaling by comparing THP-1 monocytes^23^ that were non-primed, or primed for 24 h with either 1,25(OH)_2_D_3_ or LPS, followed by stimulation with 1,25(OH)_2_D_3_, LPS, or both in combination. This immune challenge, affects the epigenomic and transcriptomic landscapes of human monocytes, which are the most vitamin D-responsive immune cell types^24^. Our findings demonstrate that exposure to an immune challenge, such as bacterial components like LPS acting as pathogen-associated molecular patterns, substantially amplifies the ability of 1,25(OH)_2_D_3_ to modulate both the epigenome and transcriptome. This suggests that immune activation sensitizes monocytes to the regulatory effects of vitamin D, potentially enhancing its immunomodulatory capacity during infections.

## Materials and methods

### Cell culture

**Fig. 1:**
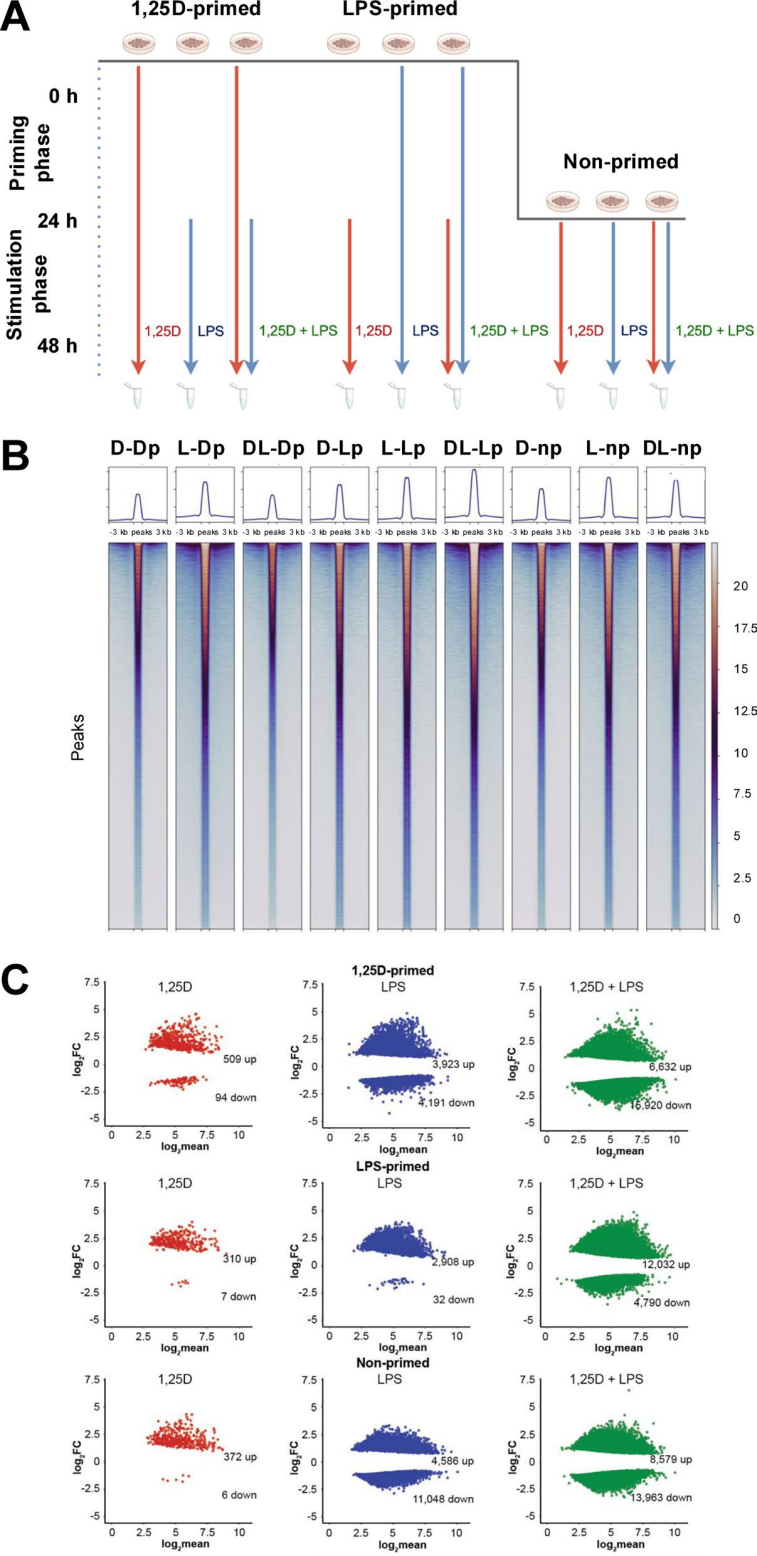
Measuring Chromatin Accessibility. Schematic overview of the experimental design (**A**). A heatmap of ATAC-seq peak intensities illustrates global changes in chromatin accessibility of all 140,727 consensus peaks following stimulation with 1,25(OH)_2_D_3_ (1,25D), LPS, or both in THP-1 cells that were primed for 24 h with 1,25(OH)_2_D_3_ (Dp), LPS (Lp), or not (np) (**B**). MA plots display differentially accessible chromatin regions (compared to solvent control), highlighting those significantly more or less accessible (FDR < 0.05) in response to each treatment (**C**).

THP-1 cells were originally derived from the peripheral blood of a 1-year-old male patient with acute monocytic leukemia and were obtained from the American Type Culture Collection (ATCC, TIB-202™)^23^. They are a well-established and physiologically relevant model for studying 1,25(OH)_2_D_3_-mediated processes, including innate immunity and cellular growth^25^. The cells were cultured in RPMI 1640 medium supplemented with 10% FBS (fetal bovine serum), 2 mM L-glutamine, 0.1 mg/ml streptomycin, and 100 U/ml penicillin, and maintained at 37 °C in a humidified incubator with 95% air and 5% CO_2_. Prior to stimulation, cells were seeded at a density of 0.5 × 10^6^ cells/ml and incubated overnight in phenol red-free medium supplemented with charcoal-stripped FBS. Cells were either left unprimed or primed for 24 h with 10 nM 1,25(OH)_2_D_3_ (dissolved in ethanol [EtOH]) or 100 ng/ml LPS (dissolved in dimethyl sulfoxide [DMSO]) (both from Sigma-Aldrich). Subsequently, all three conditions were treated for an additional 24 h with 10 nM 1,25(OH)_2_D_3_, 100 ng/ml LPS, or solvent (0.1% EtOH or 0.1% DMSO) (Fig. 1A). In the end, all samples contained 0.1% EtOH and 0.1% DMSO. All stimulation experiments were conducted in three biological replicates.

### Epigenome and transcriptome library preparation

For ATAC-seq (Assay for Transposase Accessible Chromatin with high-throughput sequencing), nuclei were isolated from 1.25 × 10^6^ cells using Buffer A (15 mM Tris-HCl, pH 8.0; 15 mM NaCl; 60 mM KCl; 1 mM EDTA; 0.5 mM EGTA; 0.5 mM spermidine; 1% protease inhibitor) supplemented with 0.04% IGEPAL (octylphenoxypolyethoxyethanol, Sigma-Aldrich). A total of 100,000 nuclei were subjected to transposition using a Tn5 transposase reaction mix (Illumina), containing Tn5 transposase, 0.01% digitonin, and 0.1% Tween-20. The reaction was incubated for 30 min at 37 °C in a Thermomixer. Following purification with the DNA Clean & Concentrator-5 Kit (Zymo Research), the ATAC-seq libraries were amplified *via* PCR, purified, and size-selected using SPRISelect purification beads (Beckman Coulter). Library quality was assessed with a Bioanalyzer (Agilent High-Sensitivity DNA Kit), and the quantified, indexed libraries were pooled for sequencing.

For RNA-seq (RNA sequencing), cells were preserved in TRIzol (1 ml per 5 × 10^6^ cells; Zymo Research) at −80 °C overnight before RNA extraction. Total RNA was isolated using the High Pure RNA Isolation Kit (Roche) with minor modifications. Chloroform (1:5 ratio) was added to thawed TRIzol samples to induce phase separation. Phase-lock gel heavy tubes (5Prime) were used to efficiently separate proteins into the organic phase, DNA at the interface, and RNA in the aqueous phase. RNA quality and quantity were assessed using a Bioanalyzer (Agilent 2100; RNA Nano 6000 Assay), ensuring a RIN (RNA integrity number) > 8. Ribosomal RNA (rRNA) was depleted using the NEBNext rRNA Depletion Kit, and complementary DNA (cDNA) libraries were prepared from the rRNA-depleted RNA using the NEBNext Ultra II Directional RNA Library Prep Kit for Illumina, following the manufacturer’s protocol. Library quality was confirmed using the Agilent DNA 1000 Chip, and indexed libraries were pooled prior to sequencing.

Both ATAC-seq and RNA-seq libraries were sequenced on an Illumina NextSeq500 system (Illumina) at 75 bp read length using standard protocols at the Gene Core facility of the EMBL (Heidelberg, Germany).

### Epigenome analysis

Quality control of the sequencing data was performed using FastQC (version 0.12.0; www.bioinformatics.babraham.ac.uk/projects/fastqc*).* Paired-end reads were aligned to the GRCh38 reference genome (GENCODE version 43) using the STAR^26^ aligner (version 2.7.2a) with default parameters unless otherwise specified. Mapped read counts are listed in **Supplementary Table ****S1**** online**. Reads mapping to ENCODE-identified blacklisted regions and low-quality reads (mapping quality score < 2) were filtered out using the samtools view function with the -q 2 parameter. Duplicate reads were identified and marked using the samtools markdup function to minimize PCR bias. Peak calling was conducted with MACS3^27^ (version 3.0.1) using the callpeak function with the following parameters: -f BAM --verbose 3 --shift 75 –extsize 150 -g hs -q 0.01. These settings enabled the identification of high-confidence peaks representing accessible chromatin regions, while retaining only one read at each exact genomic location. The overall structure of the dataset was explored using principal component analysis (PCA) via the dba.plotPCA function from the DiffBind R package. Mean Average (MA) plots were generated with ggplot2 (version 3.5.1; https://ggplot2.tidyverse.org). Heatmap for scores associated with genomic regions were generated using deepTools^28^ (version 3.5.6). The genome browser IGV (Integrative Genomics Viewer)^29,30^ was used to display regions of accessible chromatin *via* ATAC-seq peaks.

### Differential chromatin accessibility analysis

Differential chromatin accessibility analysis was performed in R (version 4.4.0) on macOS 12.6 using the DiffBind package (version 3.14.0). Consensus peak summits were generated using the dba.count function with the summits parameter set to 75, and overlapping summits were merged. This resulted in a consensus set of 140,727 peak summits (**Supplementary Table ****S2**** online**). Peaks were annotated with the ChIPseeker package^31^ (version 1.40.0), in conjunction with the TxDb.Hsapiens.UCSC.hg38.knownGene R package (version 3.18.0). Differential accessibility was assessed through pairwise comparisons of treatment groups (1,25(OH)_2_D_3_, LPS, and 1,25(OH)_2_D_3_/LPS) versus the control group, as well as between 1,25(OH)_2_D_3_ or LPS and the combined 1,25(OH)_2_D_3_/LPS treatment. Analyses were performed using DESeq2 functions integrated within DiffBind, applying the Wald test to estimate standard errors of the log₂ fold change (log₂FC) and test for significance. Peaks were considered differentially accessible if they met the following criteria defined by the dba.report function: False Discovery Rate (FDR) < 0.05 calculated under the assumption that absolute log₂FC is higher than 0.3. Motif enrichment analysis for differentially accessible regions with regions size fixed at 50 bp was conducted using HOMER (Hypergeometric Optimization of Motif EnRichment) software^32^ (version 5.1).

### Transcriptome analysis

Quality control of the sequencing data was conducted using FastQC. Single-end, reverse-stranded cDNA sequence reads were aligned to the GRCh38 reference genome (GENCODE release 43) using the STAR aligner (version 2.7.2a). Read quantification was performed with FeatureCounts^33^ (version 2.0.6). Gene annotation, including Ensembl gene identifiers, gene symbols, descriptions, genomic locations, biotypes, and Entrez Gene IDs, was carried out using the Ensembl database (release 110) *via* the BiomaRt R package^34^ (version 2.58.0). Genes missing HGNC symbols or being mitochondrially encoded were removed from the dataset. Only protein-coding genes with an expression level of at least 0.5 CPM in every sample were included in the subsequent analyses.

### Differential gene expression analysis

Differential gene expression analysis was performed in R (version 4.4.0) on macOS 12.6 using the DESeq2 package^35^ (version 1.44.0). A matrix containing raw counts for the remaining 10,904 genes and a corresponding metadata table were used to create the DESeq2 dataset (**Supplementary Table ****S3**** online**). For comparison, the original FASTQ files from a published dataset of human peripheral blood mononuclear cells (PBMCs)^36^treated according to the identical protocol, were re-analyzed using the same settings.

Two statistical approaches were employed to identify differentially expressed genes.

A: Differential expression between treatment groups (1,25(OH)_2_D_3_, LPS, and 1,25(OH)_2_D_3_/LPS) and the control group was assessed using the Wald test, which estimates the standard error of the log₂FC to determine if it significantly differs from zero. Genes with an adjusted p-value (*p*_*adj*_) < 0.001 and an absolute log₂FC > 1 were considered statistically significant. Additionally, a pairwise comparison between 1,25(OH)_2_D_3_, LPS and 1,25(OH)_2_D_3_/LPS was conducted. Genes with *p*_*adj*_ < 0.05 an absolute log₂FC > 1 were considered statistically significant.

B: For each type of priming two models were constructed: a full model and a reduced model excluding samples treated with both LPS and 1,25(OH)_2_D_3_. The likelihood ratio test (LRT) was then used to compare these models, identifying genes with significant differences between the two models. Genes with *p*_*adj*_ < 0.05 were considered statistically significant in this analysis.

## Results

### Synergistic effects of 1,25(OH)_2_D_3_ and LPS Co-stimulation on chromatin accessibility

The experimental design of this study is illustrated in Fig. 1A. THP-1 cells, as three biological replicates, were either primed for 24 h with 1,25(OH)_2_D_3_, LPS, or left unprimed. Following this priming phase, the cells underwent an additional 24-hour stimulation with 1,25(OH)_2_D_3_, LPS, or a combination of both. After the stimulation period, the cells were harvested for epigenome-wide analysis using ATAC-seq and transcriptome-wide profiling *via* RNA-seq.

ATAC-seq analysis identified 140,727 accessible chromatin regions, 20,024 of which are promoter regions (distance 500 bp from gene transcription start sites (TSSs)), 44,803 are proximal enhancers (distance 0.5–10 kb from gene TSSs) and 75,900 distal enhancers (distance more than 10 kb from gene TSSs). PCA with all 140,727 consensus accessible chromatin regions (**Supplementary Table ****S2**** online**) was conducted to evaluate the effects of stimulation with 1,25(OH)_2_D_3_, LPS, or their combination on chromatin accessibility in 1,25(OH)_2_D_3_-primed, LPS-primed, and non-primed THP-1 cells (**Supplementary Figure ****S1**** online**). The analysis revealed a pronounced shift in chromatin accessibility following LPS and 1,25(OH)_2_D_3_/LPS treatment, while 1,25(OH)_2_D_3_ alone induced comparably minor changes. These observations were consistent with the ATAC-seq peak heatmap results (Fig. 1B).

MA plots further illustrate the number of statistically significant (FDR < 0.05) changes in accessible chromatin regions (Fig. 1C). The effects of each treatment, compared to solvent control, were as follows:

- 1,25(OH)_2_D_3_ treatment: 1,25(OH)_2_D_3_-primed cells: 603 regions (509 more accessible, 94 less accessible); LPS-primed cells: 317 regions (310 more accessible, 7 less accessible); non-primed cells: 378 regions (372 more accessible, 6 less accessible).
- LPS treatment: 1,25(OH)_2_D_3_-primed cells: 8,114 regions (3,923 more accessible, 4,191 less accessible); LPS-primed cells: 2,940 regions (2,908 more accessible, 32 less accessible); non-primed cells: 15,634 regions (4,586 more accessible, 11,048 less accessible).
- Combined 1,25(OH)_2_D_3_/LPS treatment: 1,25(OH)_2_D_3_-primed cells: 22,552 regions (6,632 more accessible, 15,920 less accessible); LPS-primed cells: 16,822 regions (12,032 more accessible, 4,790 less accessible); non-primed cells: 22,542 regions (8,579 more accessible, 13,963 less accessible).

**Fig. 2:**
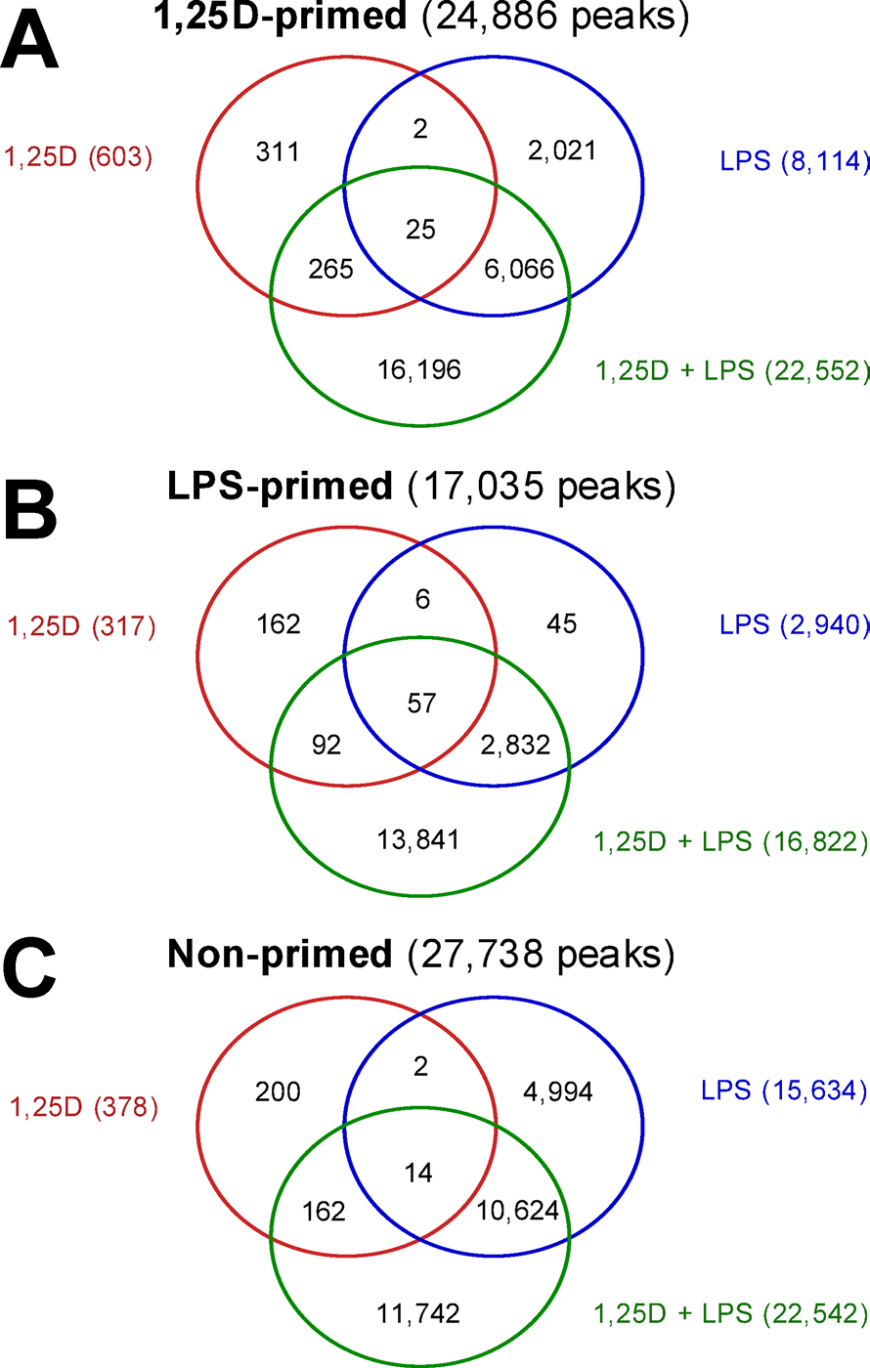
**Synergistic Effects of Co-stimulation with 1**,**25(OH)**_**2**_**D**_**3**_ **and LPS on Chromatin Accessibility.** Venn diagrams depict the overlap of differentially accessible regions following stimulation with 1,25(OH)_2_D_3_ (red), LPS (blue), or their combination (green) in THP-1 cells primed with 1,25(OH)_2_D_3_ (**A**), LPS (**B**), or left unprimed (**C**). Numbers in parentheses indicate the total number of responsive chromatin regions for each condition.

Venn diagram analyses revealed that only 25 of the 24,886 accessible chromatin regions primed by 1,25(OH)_2_D_3_ were significantly affected by all three types of stimulation (Fig. 2A). Similarly low overlap rates were observed for LPS-primed regions (57 of 17,035; Fig. 2B) and non-primed regions (14 of 27,738; Fig. 2C). In contrast, a substantial number of 1,25(OH)_2_D_3_-primed regions (16,196 of 24,886) were uniquely altered following co-stimulation with 1,25(OH)_2_D_3_ and LPS (Fig. 2A). This trend was consistent in LPS-primed regions (13,841 of 17,035; Fig. 2B) and non-primed regions (11,742 of 27,738; Fig. 2C), which also showed a high proportion of co-stimulation-specific changes.

In summary, ATAC-seq analysis emphasizes the dominant role of LPS and, more prominently, the synergistic effect of 1,25(OH)_2_D_3_/LPS co-treatment in modulating chromatin accessibility in THP-1 cells. While in each type of priming all three stimuli together affect only a minor fraction of significantly altered chromatin regions (overlap: 0.05–0.33%), a large proportion of the observed changes (42.4–81.3%) are exclusively induced by 1,25(OH)_2_D_3_/LPS co-stimulation.

### 1,25(OH)_2_D_3_/LPS Co-Stimulation broadly alters chromatin accessibility and enriches JUN/FOS motifs

**Fig. 3:**
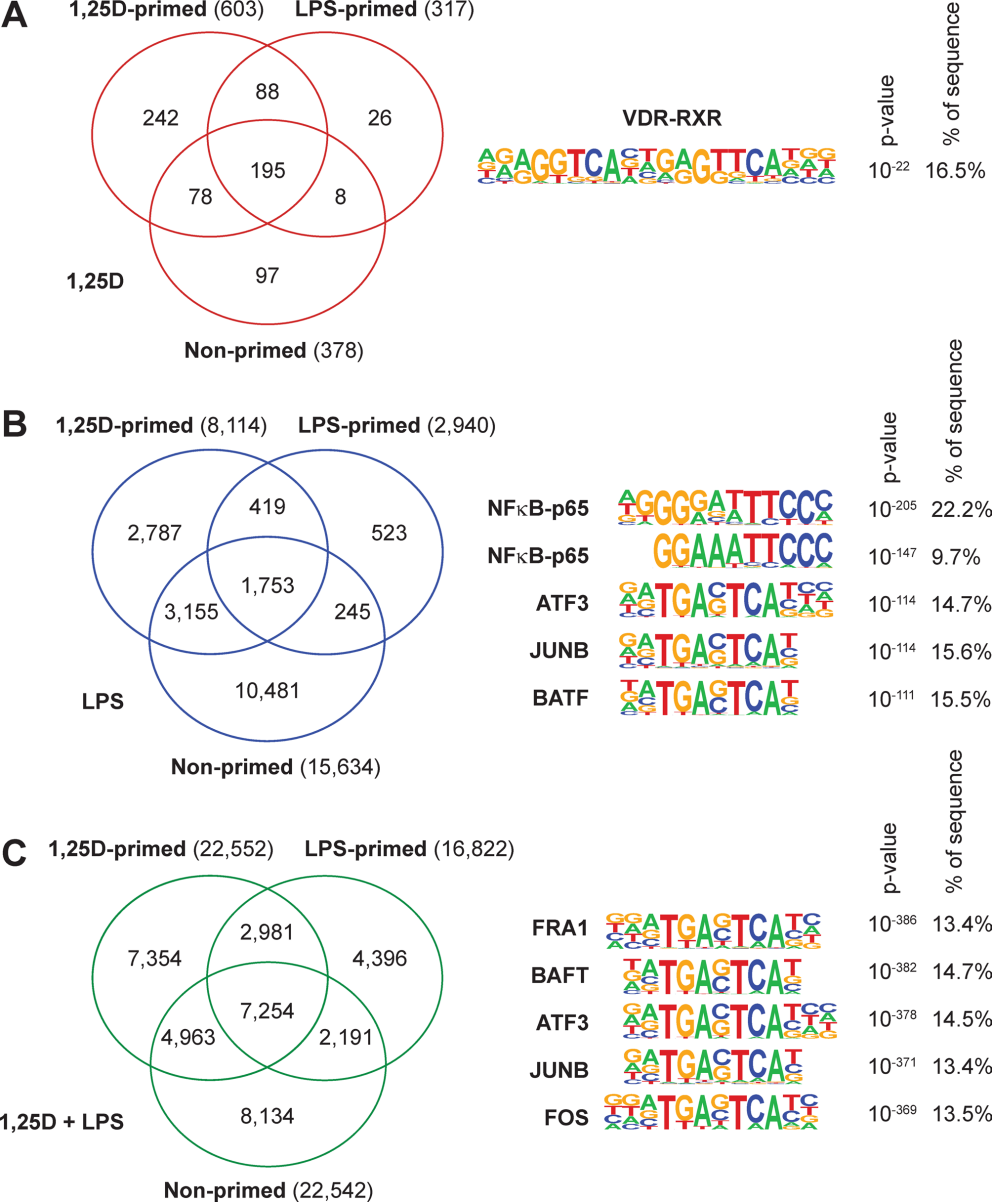
**Co-stimulation with 1**,**25(OH)**_**2**_**D**_**3**_ **and LPS Broadly Alters Chromatin Accessibility and Enriches JUN/FOS Motifs.** Venn diagrams show the overlap of differentially accessible regions in response to stimulation with 1,25(OH)_2_D_3_ (red, **A**), LPS (blue, **B**), or both (green, **C**) in THP-1 cells under different priming conditions. Motif enrichment analysis (HOMER) of the overlapping regions reveals significant enrichment of JUN/FOS motifs, indicating potential involvement of AP1 transcription factors.

Comparison of the three priming models in THP-1 cells, stimulated with 1,25(OH)_2_D_3_, LPS, or their combination, revealed 734 chromatin target regions responsive to 1,25(OH)_2_D_3_ (Fig. 3A), 19,363 regions reacting to LPS (Fig. 3B), and 37,273 targets altered by co-stimulation (Fig. 3C). These findings further highlight that the combined treatment affects substantially more chromatin regions than either stimulus alone. This trend also holds for the number of shared target regions across the three priming conditions: 195 for 1,25(OH)_2_D_3_, 1,753 for LPS, and 7,254 for the combined stimulation.

Representative genome browser tracks illustrate specific examples of stimulus-dominated gene regulation: the enhancer or TSS regions of *ASAP2* (ArfGAP with SH3 domain, ankyrin repeat and PH domain 2) predominantly respond to 1,25(OH)_2_D_3_ (**Supplementary Figure ****S2****A online**), those of *IL4I1* (interleukin 4 induced 1) are mainly driven by LPS (**Supplementary Figure ****S2****B online**) and those of *G0S2* (G0/G1 switch 2) are primarily regulated by the combined treatment (**Supplementary Figure ****S2****C online**).

To identify transcription factor binding motifs enriched in the overlapping chromatin regions, HOMER analysis was performed. For the 1,25(OH)_2_D_3_-responsive regions, the only highly significantly enriched motif was that of the VDR-RXR (retinoid X receptor) heterodimer (Fig. 3A). In contrast, LPS-responsive regions were enriched for NFκB motifs, followed by binding sites for JUN/FOS family members, including ATF3 (activating transcription factor 3), JUNB (JunB proto-oncogene), and BATF (basic leucine zipper ATF-like transcription factor) (Fig. 3B). Interestingly, the most enriched motifs in the regions responsive to 1,25(OH)_2_D_3_/LPS co-stimulation were exclusively from the JUN/FOS family, such as FOSL1 (FOS-like 1, also called FRA1), BATF, ATF3, JUNB, and FOS (Fos proto-oncogene) (Fig. 3C).

Taken together, combined stimulation with 1,25(OH)_2_D_3_ and LPS affects a substantially greater number of chromatin regions in THP-1 cells than either treatment alone. Motif analysis reveals that co-stimulation predominantly enriches JUN/FOS transcription factor binding sites, highlighting a distinct regulatory signature.

### Distinct transcriptional programs induced by Co-stimulation with 1,25(OH)_2_D_3_ and LPS

**Fig. 4:**
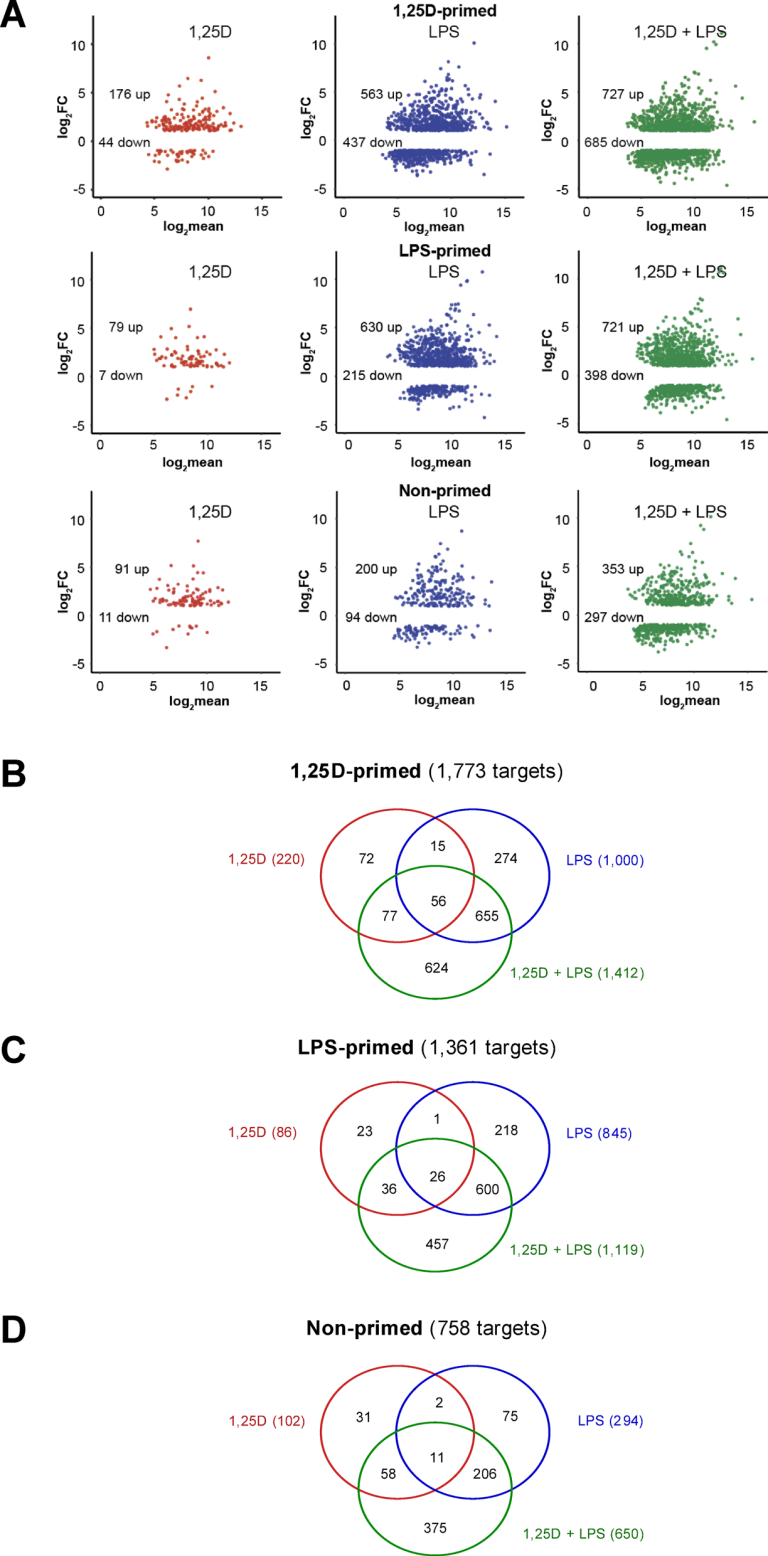
**Distinct Transcriptional Programs Induced by Co-stimulation with 1**,**25(OH)**_**2**_**D**_**3**_ **and LPS.** MA plots illustrate gene expression changes following stimulation with 1,25(OH)_2_D_3_ (red), LPS (blue), or both (green) in THP-1 cells primed with 1,25(OH)₂D₃, LPS, or left unprimed (**A**). The number of significantly upregulated and downregulated genes is indicated. Venn diagrams show the overlap of significantly regulated genes in cells primed with 1,25(OH)_2_D_3_ (**B**), LPS (**C**), or not primed (**D**), upon subsequent stimulation.

To assess the functional consequences of epigenomic changes induced by 1,25(OH)_2_D_3_, LPS, and their combination, transcriptome profiling was performed using RNA-seq in THP-1 cells treated under the same conditions as those used for ATAC-seq (Fig. 1A). The analysis focused on 10,904 protein-coding genes (**Supplementary Table ****S3**** online**). PCA confirmed a marked transcriptional response to 1,25(OH)_2_D_3_, LPS, and their combined stimulation in 1,25(OH)_2_D_3_-primed, LPS-primed, and non-primed cells (**Supplementary Figure ****S3**** online**). MA plots further illustrated the extent of differential gene expression, highlighting genes with statistically significant changes (FDR < 0.001; Fig. 4A). The number of differentially expressed genes for each condition was as follows:

1,25(OH)_2_D_3_ treatment: 1,25(OH)_2_D_3_-primed: 220 genes (176 upregulated, 44 downregulated); LPS-primed: 86 genes (79 upregulated, 7 downregulated); non-primed: 102 genes (91 upregulated, 11 downregulated).

LPS treatment: 1,25(OH)_2_D_3_-primed: 1,000 genes (563 upregulated, 437 downregulated); LPS-primed: 845 genes (630 upregulated, 215 downregulated); non-primed: 294 genes (200 upregulated, 94 downregulated).

Combined 1,25(OH)_2_D_3_ and LPS treatment: 1,25(OH)_2_D_3_-primed: 1,412 genes (727 upregulated, 685 downregulated); LPS-primed: 1,119 genes (721 upregulated, 398 downregulated); non-primed: 650 genes (353 upregulated, 297 downregulated).

Venn diagram analyses revealed limited overlap among genes affected by all three stimulation conditions. Specifically, only 56 of the 1,773 1,25(OH)_2_D_3_-primed target genes were commonly regulated across all treatments (Fig. 4B). Similarly, overlap was minimal in LPS-primed (26 of 1,361; Fig. 4C) and non-primed cells (11 of 758; Fig. 4D). In contrast, a substantial proportion of genes were uniquely responsive to the combined 1,25(OH)_2_D_3_/LPS treatment: 624 of 1,773 in 1,25(OH)_2_D_3_-primed (Fig. 4B), 457 of 1,361 in LPS-primed (Fig. 4C), and 375 of 758 in non-primed cells (Fig. 4D). These findings highlight a strong co-stimulation-specific transcriptional signature. The overall structure of the transcriptomic response to the different stimuli is illustrated by hierarchical clustering of all 2,086 responding genes (**Supplementary Figure ****S4**** online**).

To validate the results of the transcriptome analysis, we used a dataset from an in vitro experiment in human PBMCs that followed the same treatment protocol^36^. This independently performed experiment confirmed 13.7–24.8% of the identified target genes (**Supplementary Table ****S3**** online**). The confirmation rate would likely be higher if PBMCs expressed more than 79% of the target genes identified in THP-1 cells. It should be noted that PBMCs represent a heterogeneous mixture of immune cell types, whereas THP-1 cells constitute a homogeneous monocytic model. Consequently, it is not surprising that, unlike in THP-1 cells, the majority of target genes in PBMCs are downregulated in response to the different stimuli.

In summary, the transcriptomic analysis yielded results largely consistent with the epigenomic findings, albeit with approximately tenfold fewer genes showing a response to the different types of stimulation compared to the number of affected chromatin regions.

### Distinct gene regulation by Co-Stimulation with 1,25(OH)_2_D_3_ and LPS reveals novel targets linked to immune cell differentiation

**Fig. 5:**
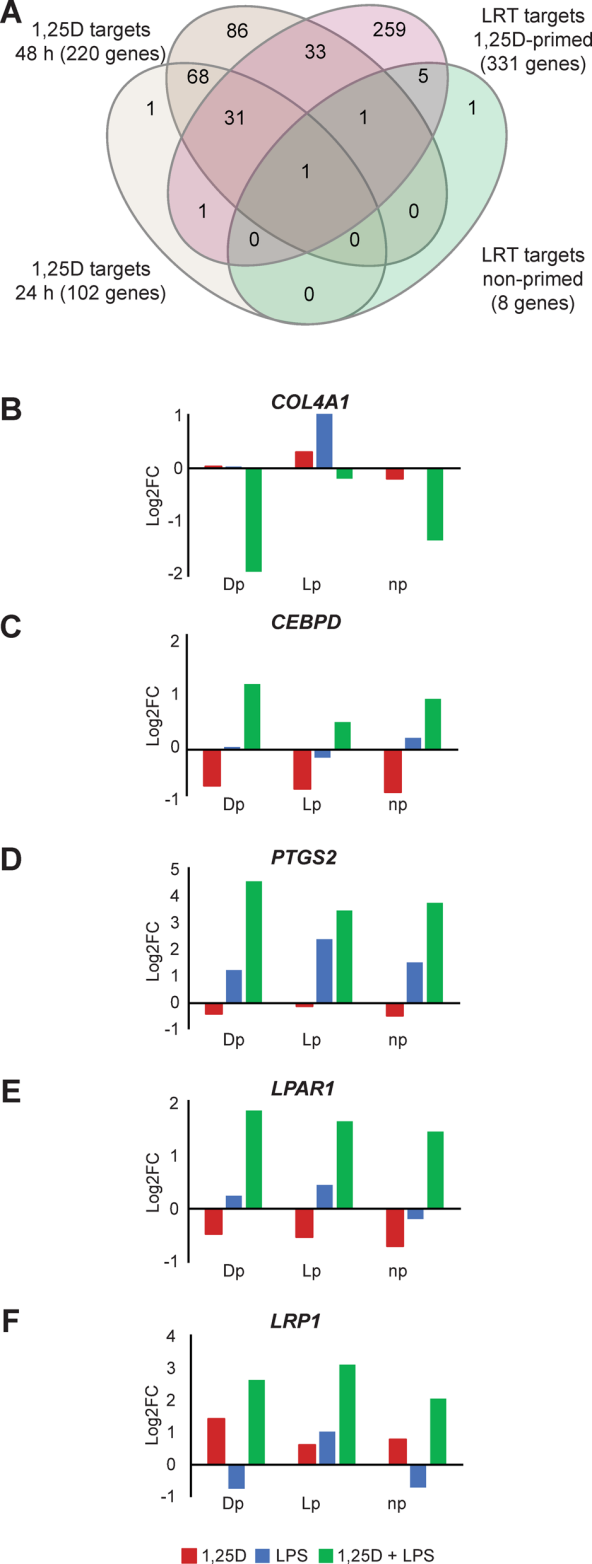
**Synergistic Gene Regulation by Co-Stimulation with 1**,**25(OH)**_**2**_**D**_**3**_ **and LPS.** Venn diagram illustrating the overlap between classical vitamin D target genes (after 24 and 48 h of stimulation) and genes identified as synergistically regulated *via* LRT analysis (**A**). Bar plots show expression changes in representative synergistically regulated genes: *COL4A1* (**B**), *CEBPD* (**C**), *PTGS2* (**D**), *LPAR1* (**E**), and *LRP1* (**F**), comparing single and combined treatments.

To identify genes exhibiting a synergistic response to co-stimulation with 1,25(OH)_2_D_3_ and LPS, LRT analyses were performed. This approach assesses whether the combined treatment elicits a significantly different effect on gene expression (FDR < 0.05) than the additive effects of the individual treatments. Strikingly, under 1,25(OH)_2_D_3_-primed conditions, 331 genes showed a significantly distinct response. In contrast, no such genes were identified under LPS-primed conditions, and only 8 were detected in non-primed cells. From the latter genes 7 overlapped with those identified under 1,25(OH)_2_D_3_-primed conditions (Fig. 5A). Interestingly, 33 and 34 of the synergistically regulated genes had previously been classified as vitamin D targets after 24 and 48 h of 1,25(OH)_2_D_3_ stimulation, respectively. This leaves 264 genes as newly identified targets, not responsive to 1,25(OH)_2_D_3_ alone.

Comparison of the functional profiles of the 264 newly identified synergistic target genes with the 222 classical vitamin D targets revealed notable differences. Gene ontology analysis using EnrichR^37^ analysis (**Supplementary Table ****S4**** online**) indicated that the synergistic targets are primarily associated with monocyte and T cell differentiation, whereas the classical targets are mainly involved in mediating the inflammatory response. Major target genes mediating this function are CD4 (CD4 molecule), CD74, CAMK4 (calcium/calmodulin dependent protein kinase IV), HLA-DRB1 (major histocompatibility complex, class II, DR beta 1), MYC (MYC proto-oncogene, BHLH transcription factor), MYH9 (myosin heavy chain 9), PDE1B (phosphodiesterase 1B), RUNX1 (RUNX family transcription factor 1), RUNX3 and ZBTB7B (zinc finger and BTB domain containing 7B).

As an alternative to the LRT approach, we compared gene expression profiles of co-treated cells with those treated individually with either 1,25(OH)_2_D_3_ (1,920 differentially expressed genes) or LPS (436 genes) (**Supplementary Figure ****S5**** online**). This analysis revealed an overlap of 239 genes, 52 of which were also identified by the LRT analysis, further supporting their synergistic regulation.

To improve the visibility of key transcriptional targets, we visualized the expression profiles of selected genes identified by LRT analysis across all treatment conditions. These include *COL4A1* (collagen type IV alpha 1 chain), which is strongly downregulated upon co-treatment (Fig. 5B). In contrast, genes such as *CEBPD* (CCAAT/enhancer-binding protein delta, Fig. 5C**)**, *PTGS2* (prostaglandin-endoperoxide synthase 2, Fig. 5D), and *LPAR1* (lysophosphatidic acid receptor 1, Fig. 5E) are downregulated by 1,25(OH)_2_D_3_ alone but upregulated under co-stimulation. Additionally, the *LRP1* (LDL receptor-related protein 1) gene shows an upregulation with 1,25(OH)_2_D_3_ alone that is further enhanced by co-treatment, indicating a true synergistic effect (Fig. 5F). These genes are functionally linked to extracellular matrix remodeling, inflammation, and lipid signaling, processes relevant to innate immune activation and tissue homeostasis. Notably, most of the 52 genes identified by LRT analysis exhibited enhanced expression upon combined 1,25(OH)₂D₃ and LPS stimulation, consistent with a synergistic transcriptional program.

In addition, we analyzed the expression of genes encoding members of the AP1 transcription factor family that responded to 1,25(OH)_2_D_3_, LPS, and/or their combination. Specifically, we focused on the genes encoding for ATF3, FOS, FOSL1, JUN, and JUNB, which were also identified as enriched motifs in our ATAC-seq analysis (**Supplementary Figure S6 online**). Among these, ATF3 showed the strongest induction, particularly in response to LPS alone or in combination with 1,25(OH)_2_D_3_, whereas the other genes of the AP1 family exhibited more moderate expression changes. These findings suggest that ATF3 may play a key role in mediating the observed alterations in chromatin accessibility and downstream transcriptional responses.

Taken together, co-stimulation with 1,25(OH)_2_D_3_ and LPS under 1,25(OH)_2_D_3_-primed conditions revealed 331 genes with synergistic expression changes, including 264 newly identified targets not responsive to either treatment alone. Functional analysis showed that these synergistic targets are primarily involved in monocyte and T cell differentiation, distinguishing them from classical vitamin D targets associated with inflammatory responses.

## Discussion

This study demonstrates that co-stimulation with 1,25(OH)_2_D_3_ and LPS in THP-1 monocytes induces a distinct epigenomic and transcriptomic program that far exceeds the effects of either treatment alone. The integration of ATAC-seq and RNA-seq data revealed that the treatment with 1,25(OH)_2_D_3_ and LPS, alone and in combination, alters chromatin accessibility at over 41,500 genomic regions and significantly regulates the expression of more than 2,000 genes, including a subset of 331 showing clear synergistic behavior under 1,25(OH)_2_D_3_-primed conditions. These synergistically regulated genes, many of which were previously unrecognized as vitamin D targets, were functionally enriched in pathways related to monocyte and T cell differentiation, rather than inflammation, suggesting that vitamin D may preferentially prime innate immune cells for differentiation rather than inflammatory activation when confronted with a bacterial stimulus.

Mechanistically, the data suggest that co-stimulation leads to the recruitment of a distinct set of transcription factors. Motif enrichment analysis revealed a shift from canonical VDR-RXR binding in 1,25(OH)_2_D_3_-treated cells and NFκB motifs in LPS-treated cells to a dominant enrichment of motifs of JUN, FOS and other members of the AP1 transcription family under co-stimulation. ATF3 emerges as a key regulatory factor, showing strong upregulation in response to LPS alone or in combination with 1,25(OH)_2_D_3_. This implies that the heterodimeric AP1 transcription factor complexes could be central integrators of the vitamin D and LPS signaling pathways. This may lead to the formation of novel transcriptional complexes or chromatin landscapes that enable the observed synergistic gene regulation. Such cooperation could involve chromatin priming by VDR that facilitates subsequent AP1 binding upon LPS stimulation, or vice versa. Interestingly, the interaction of VDR and AP1 signaling had been described first more than 30 years ago^38^.

A striking observation is the disproportion between chromatin and transcriptional changes. From the 41,522 affected chromatin regions 2,692 are promoters, while 13,895 are proximal enhancer and 24,935 distal enhancers. The rather low number of affected promoters may relate to the 2,086 regulated genes. The large number of affected enhancer regions emphasizes the complex, multi-layered regulation of gene expression, where chromatin remodeling is necessary but not always sufficient for transcriptional activation^39^. It also suggests the presence of additional regulatory layers such as post-transcriptional control, enhancer-promoter looping, or protein-level modulation that remain to be explored^40^.

From a clinical perspective, these findings have important implications for both infectious and inflammatory diseases. Notably, 331 genes primed by 1,25(OH)_2_D_3_ passed the LRT analysis, whereas none of the LPS-primed genes met this criterion. This suggests that pre-existing vitamin D signaling, achieved through supplementation prior to infection, may be crucial for eliciting a robust and coordinated immune response. These results are consistent with previous observations in peripheral blood mononuclear cells, reinforcing the importance of vitamin D_3_ in immune priming^36^.

Vitamin D deficiency is widespread and has been associated with increased susceptibility to infections and poor outcomes in inflammatory conditions^41^. Our results suggest that sufficient vitamin D levels may enable immune cells to mount a more finely tuned response to bacterial infection: one that favors immune cell differentiation and resolution rather than excessive inflammation. This could be particularly relevant in diseases like sepsis, tuberculosis, or chronic inflammatory disorders, where immune modulation rather than suppression is often the therapeutic goal^42^. Moreover, the identification of a large set of co-stimulation-specific genes opens the door to discovering new biomarkers or therapeutic targets influenced by the vitamin D–LPS axis.

Nevertheless, several limitations must be acknowledged. First, the study was conducted in THP-1 cells, an immortalized monocytic cell line^43^which, although widely used, does not fully recapitulate the complexity and heterogeneity of primary human monocytes or tissue-resident macrophages. This may explain why only approximately one in five target genes could be confirmed in an identical experiment performed in human PBMCs. Moreover, PBMCs are a mixed cell population containing not only monocytes but also T cells, B cells, and NK (natural killer) cells. Second, we focused on a 48-hour timeframe; earlier or later time points might reveal additional dynamic regulatory changes. Third, the study does not yet establish causality between JUN/FOS binding and gene regulation, nor does it address how chromatin changes translate into functional immune phenotypes. Further validation using ChIP-seq (chromatin immunoprecipitation sequencing) for AP1 transcription factor family members, such as ATF3, functional assays, and studies in primary cells or in vivo models will be essential to extend these findings.

In conclusion, our work uncovers a synergistic interplay between 1,25(OH)_2_D_3_ and LPS that reprograms the epigenetic and transcriptional landscape of monocytes in a manner distinct from either stimulus alone. The identification of a large set of co-stimulation-specific genes, enriched in differentiation-related pathways and marked by AP1 motifs, suggests a unique regulatory mechanism with potential immunomodulatory and therapeutic relevance. These findings contribute to a deeper understanding of how micronutrient signaling integrates with innate immune cues to shape cellular identity and function.

## Electronic supplementary material

Below is the link to the electronic supplementary material.

Supplementary Material 1

Supplementary Material 2

Supplementary Material 3

Supplementary Material 4

Supplementary Material 5

## Data Availability

The raw sequencing data (FASTQ files) are available at the Gene Expression Omnibus (GEO, www.ncbi.nlm.nih.gov/geo) under accession numbers GSE293963 and GSE293966.

## Abbreviations

1,25(OH)_2_D_3_or 125D: 1α,25-dihydroxyvitamin D_3_
AP1: activator protein 1
ASAP2: ArfGAP with SH3 domain, ankyrin repeat and PH domain 2
ATAC-seq: Assay for Transposase Accessible Chromatin with high-throughput sequencing
ATF3: activating transcription factor 3
BATF: basic leucine zipper ATF-like transcription factor
CAMK4: calcium/calmodulin dependent protein kinase IV
ChIP-seq: chromatin immunoprecipitation sequencing
CD4: CD4 molecule
CD4 molecule: CCAAT/enhancer-binding protein delta
COL4A1: collagen type IV alpha 1 chain
CPM: counts per million
CREB1: cAMP response element-binding protein 1
FBS: fetal bovine serum
FC: fold change
FDR: false discovery rate
FOS: Fos proto-oncogene, AP1 transcription factor subunit
FOSL1: FOS like 1, AP1 transcription factor subunit, also called FRA1
G0S2: G0/G1 switch 2
HLA-DRB1: major histocompatibility complex, class II, DR beta 1
IGV: Integrative Genomics Viewer
IL4I1: interleukin 4 induced 1
JUN: Jun proto-oncogene, AP1 transcription factor subunit
JUNB: JunB proto-oncogene, AP1 transcription factor subunit
LPAR1: lysophosphatidic acid receptor 1
LPS: lipopolysaccharide
LRP1: LDL receptor-related protein 1
LRT: likelihood ratio test
MAPK: mitogen-activated protein kinase
MYC: MYC proto-oncogene, BHLH transcription factor
MYH9: myosin heavy chain 9
NFκB: nuclear factor kappa B
PBMC: peripheral blood mononuclear cell
PCA: principal component analysis
PDE1B: phosphodiesterase 1B
PTGS2: prostaglandin-endoperoxide synthase 2
RIN: RNA integrity number
RNA-seq: RNA sequencing
RUNX1: RUNX family transcription factor 1
RXR: retinoid X receptor
TLR4: Toll-like receptor 4
TSS: transcription start site
VDR: vitamin D receptor
ZBTB7B: zinc finger and BTB domain containing 7B
