# Supplementary material for: Vitamin D and lipopolysaccharide jointly induce a distinct epigenetic and transcriptional program in human monocytes
Source: Sci Rep. 2025 Jul 28;15:27480. doi: 10.1038/s41598-025-10921-2 (PMC12304155; doi:10.1038/s41598-025-10921-2)
Supplement: Supplementary file 5 — Supplementary Material 5 [file 41598_2025_10921_MOESM5_ESM.pdf]

SUPPLEMENTARY DATA

SUPPLEMENTARY TABLES

Table S1. Read Alignment Statistics. The number of uniquely aligned reads per sample is reported for both ATAC-seq and RNA-seq datasets. Five RNA-seq samples were excluded from downstream analysis due to quality concerns. Specifically, one replicate each from the DL-Lp and DL-np treatment groups was removed due to abnormally high or low total read counts. Additionally, one replicate each from the L-Lp and solvent 24 h groups was excluded due to a low percentage of uniquely aligned reads, and one replicate from the L-np group was omitted because of an aberrant gene expression profile. Consequently, all differential expression analyses involving these treatment groups were performed using two biological replicates.

Table S2. Epigenomic Changes in THP-1 Cells. THP-1 cells were primed in triplicate for 24 hours with either 10 nM 1,25(OH)$_2$D$_3$ (Dp), 100 ng/ml LPS (Lp), or left unprimed (np), followed by stimulation for an additional 24 hours with 10 nM 1,25(OH)$_2$D$_3$ (D), 100 ng/ml LPS (L), or both (DL). ATAC-seq analysis identified 140,727 accessible chromatin regions, of which 41,522 showed significant changes in response to at least one treatment condition (highlighted in green).

Table S3. Transcriptomic Changes in THP-1 Cells. The same experimental setup as in **Table S2** was applied. RNA-seq analysis identified 10,904 expressed protein-coding genes (CPM > 0.05), of which 2,086 responded significantly to at least one treatment condition (highlighted in green). As a reference, data from an experiment performed in human PBMCs using the identical protocol indicated which target genes were confirmed in this cellular model.

Table S4. Gene Ontology Analysis of Synergistically Regulated Genes. GO enrichment analysis of 264 synergistically regulated target genes (identified *via* LRT; **Figure 5A**) and 222 classical vitamin D target genes was performed using EnrichR. Enriched biological processes are listed and ranked by adjusted p-value.

SUPPLEMENTARY FIGURES

Figure S1. ATAC-seq Sample Quality Control. PCA was applied to assess the clustering of ATAC-seq triplicates and to visualize treatment effects of 1,25(OH)$_2$D$_3$ (1,25D), LPS, or both, in comparison to solvent controls. Data are shown for 1,25(OH)$_2$D$_3$-primed (Dp), LPS-primed (Lp), and unprimed (np) THP-1 cells.

Figure S2. Representative Differential Chromatin Accessibility Regions. ATAC-seq profiles were visualized using the IGV browser. THP-1 cells were treated with 1,25(OH)$_2$D$_3$ (red), LPS (blue), 1,25(OH)$_2$D$_3$ + LPS (green), or solvent (grey), after priming with 1,25(OH)$_2$D$_3$ (Dp), LPS (Lp), or no priming (np). Treatment-responsive enhancer and TSS regions are highlighted in light grey for the vitamin D target genes *ASAP2* (**A**), *IL4I1* (**B**), and *G0S2* (**C**). Tracks represent merged data from three biological replicates.

Figure S3. RNA-seq Sample Quality Control. PCA was used to visualize the clustering of RNA-seq triplicates and treatment effects (1,25(OH)$_2$D$_3$, LPS, or both versus solvent) under 1,25(OH)$_2$D$_3$-primed (Dp), LPS-primed (Lp), and unprimed (np) conditions in THP-1 cells.

Figure S4. Transcriptional response of THP-1 cells. Hierarchical clustering was performed on all 2,086 genes that responded significantly (FDR < 0.05) to stimulation with 1,25(OH)$_2$D$_3$, LPS, or their combined treatment.

Figure S5. Synergistic Transcriptional Effects of 1,25(OH)$_2$D$_3$ and LPS. A Venn diagram illustrates the overlap of genes significantly (FDR < 0.05, absolute log$_2$FC > 1) regulated by combined treatment (1,25(OH)$_2$D$_3$ + LPS) compared to those

regulated by individual stimuli (1,25$(OH)_2D_3$ in red, LPS in blue). Genes identified as synergistically regulated by LRT analysis (FDR < 0.05) are shown in purple.

Figure S6: Differential expression of selected genes in THP-1 cells across treatment conditions. Bar plots show log_2FC in expression for AP1 family members (ATF3, FOS, FOSL1, JUN, JUNB) under 1,25$(OH)_2D_3$ (1,25D), LPS and combined stimulation. Values represent mean expression fold change relative to solvent controls.

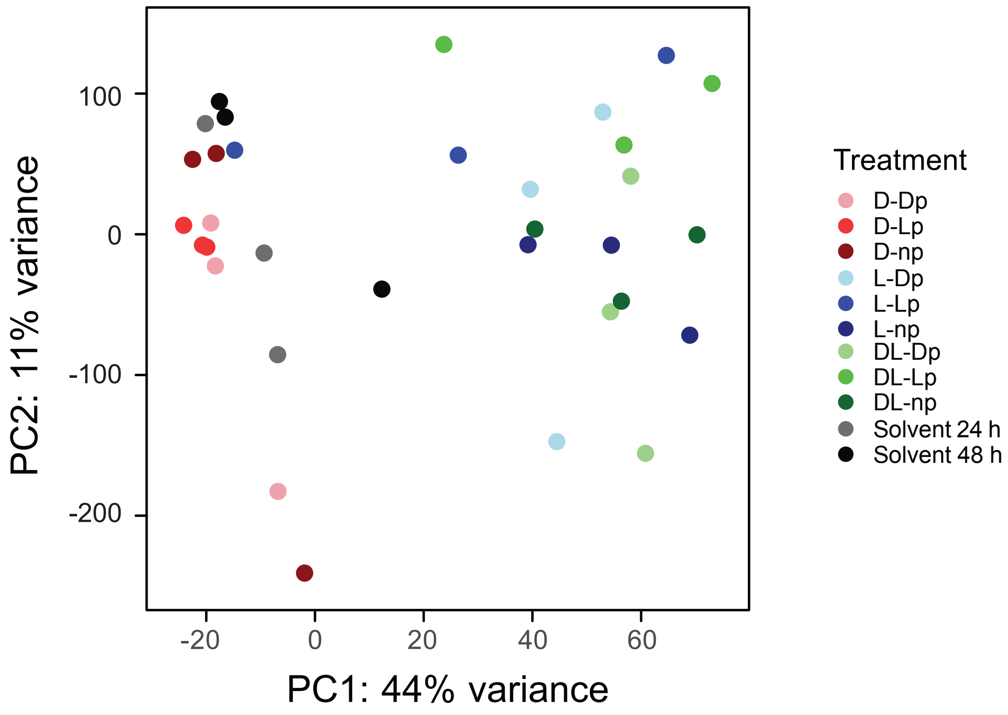
PC2: 11% variance
100
0
-100
-200
-20
0
20
40
60
PC1: 44% variance
Treatment
D-Dp
D-Lp
D-np
L-Dp
L-Lp
L-np
DL-Dp
DL-Lp
DL-np
Solvent 24 h
Solvent 48 h

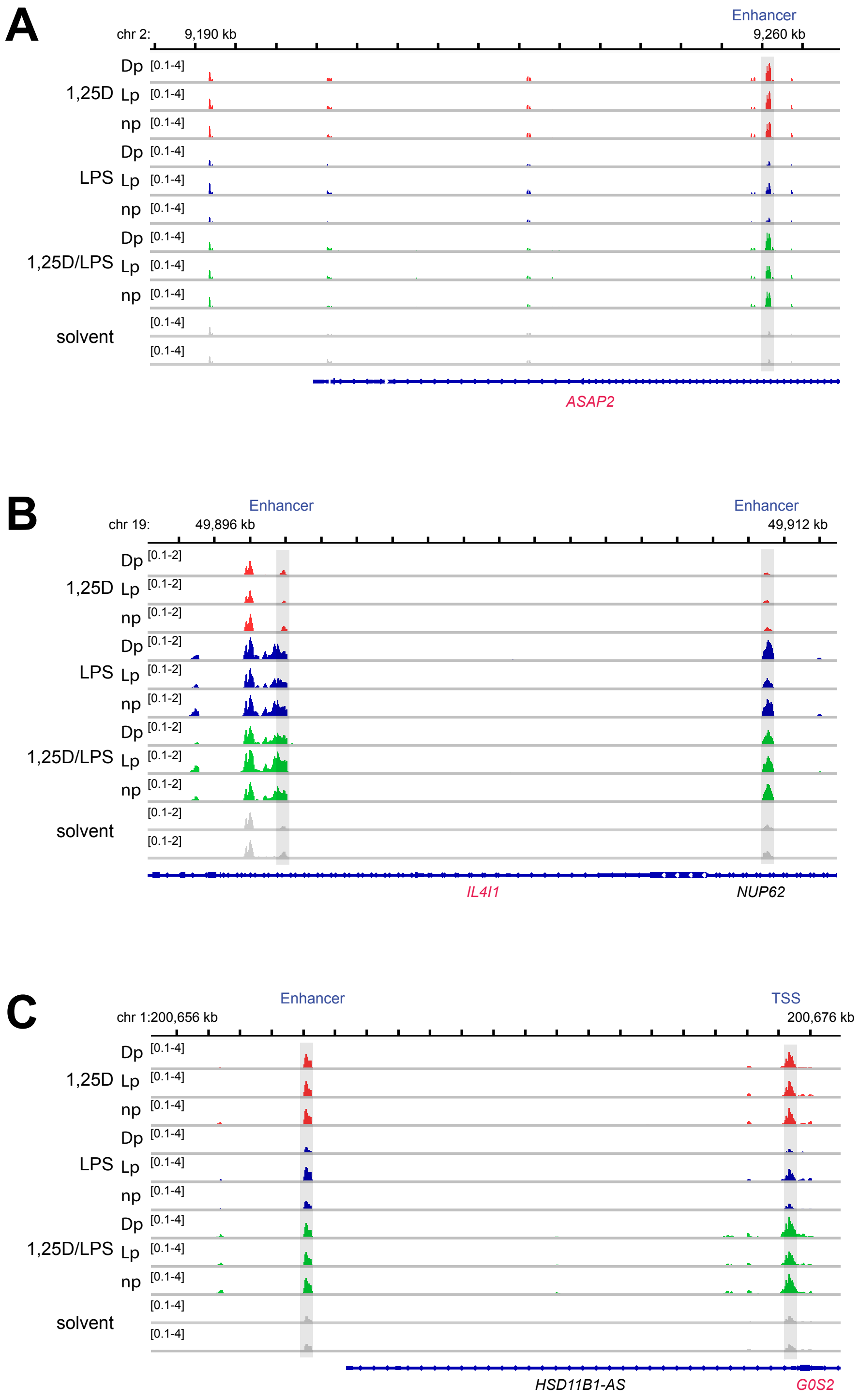
A
Enhancer
chr 2:
9,190 kb
9,260 kb
1,25D
LPS
1,25D/LPS
solvent
Dp
Lp
np
[0.1-4]
ASAP2
B
Enhancer
Enhancer
chr 19:
49,896 kb
49,912 kb
[0.1-2]
IL4I1
NUP62
C
Enhancer
TSS
chr 1:200,656 kb
200,676 kb
HSD11B1-AS
G0S2

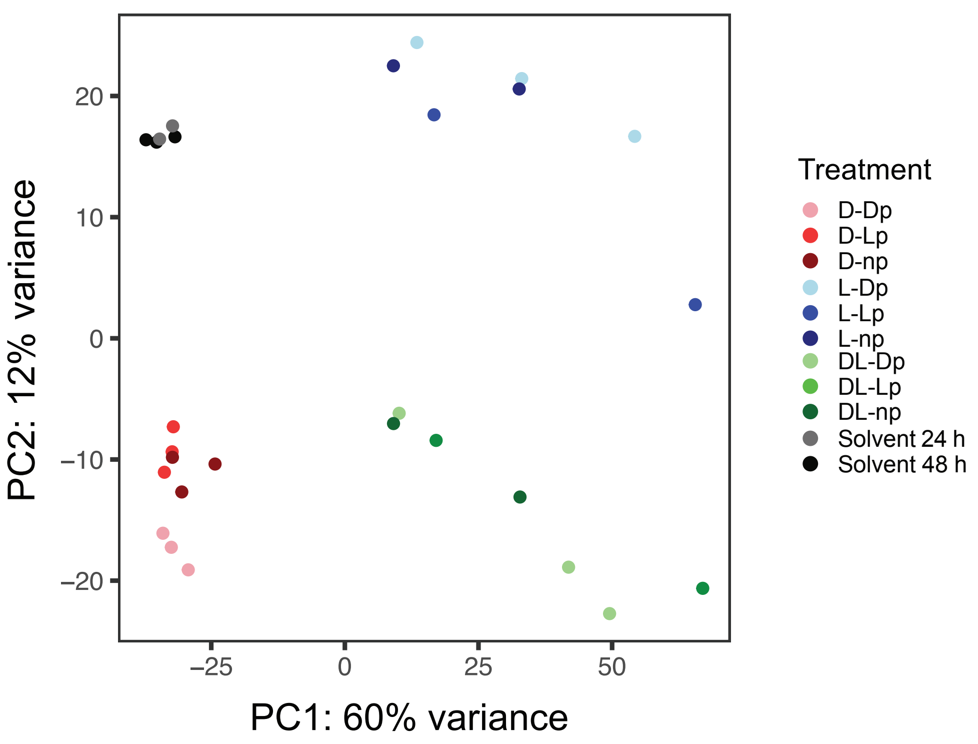
PC1: 60% variance
PC2: 12% variance
−25
0
25
50
20
10
0
−10
−20
Treatment
D-Dp
D-Lp
D-np
L-Dp
L-Lp
L-np
DL-Dp
DL-Lp
DL-np
Solvent 24 h
Solvent 48 h

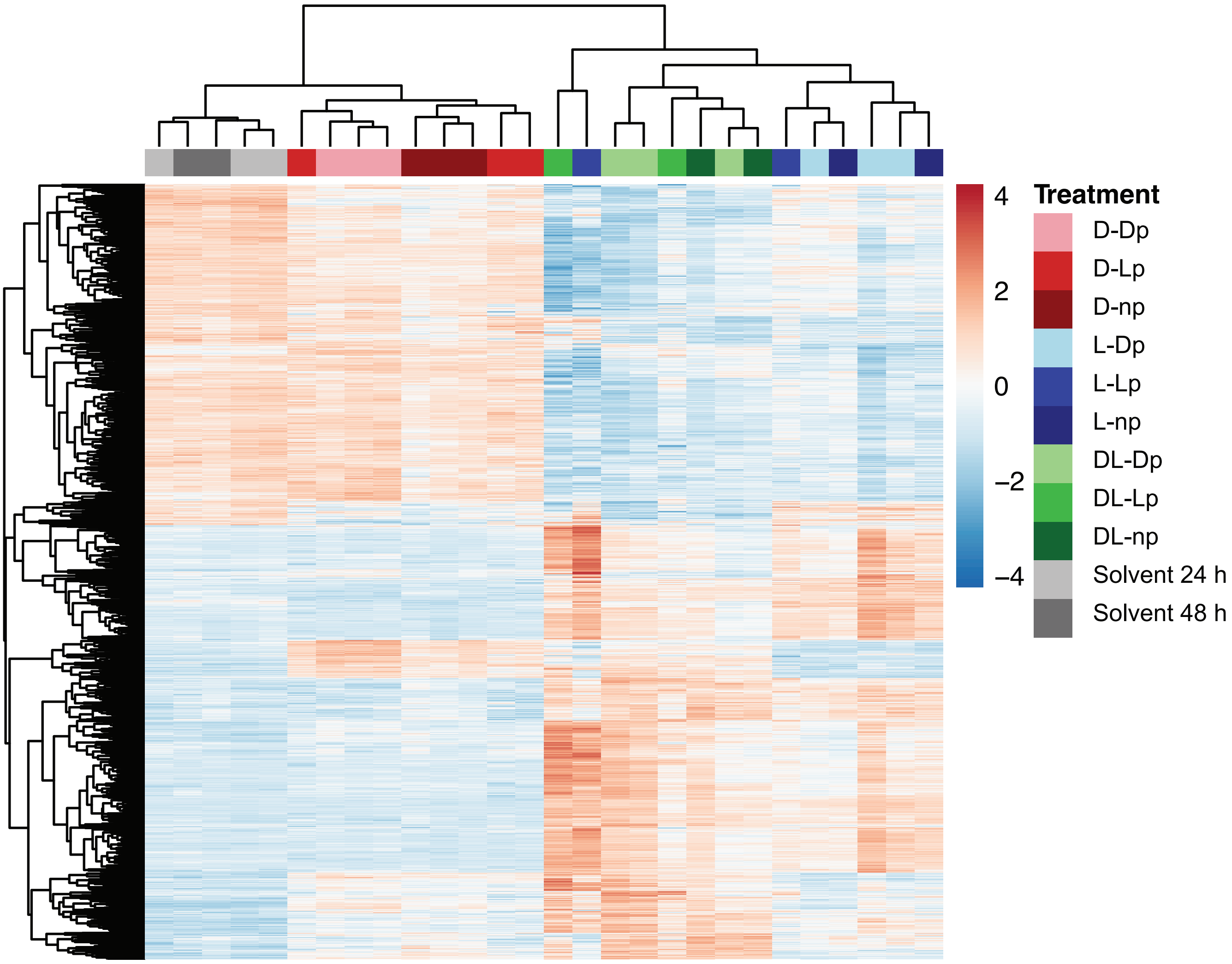
4
2
0
−2
−4
Treatment
D-Dp
D-Lp
D-np
L-Dp
L-Lp
L-np
DL-Dp
DL-Lp
DL-np
Solvent 24 h
Solvent 48 h

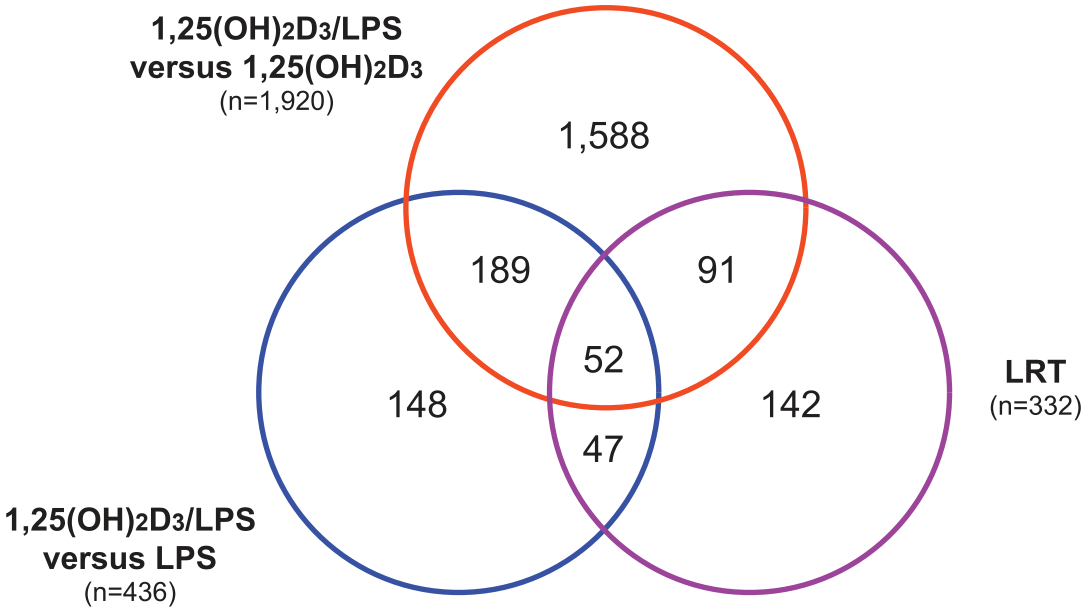
1,25(OH)2D3/LPS versus 1,25(OH)2D3
(n=1,920)
1,588
189
91
52
148
142
47
LRT
(n=332)
1,25(OH)2D3/LPS versus LPS
(n=436)

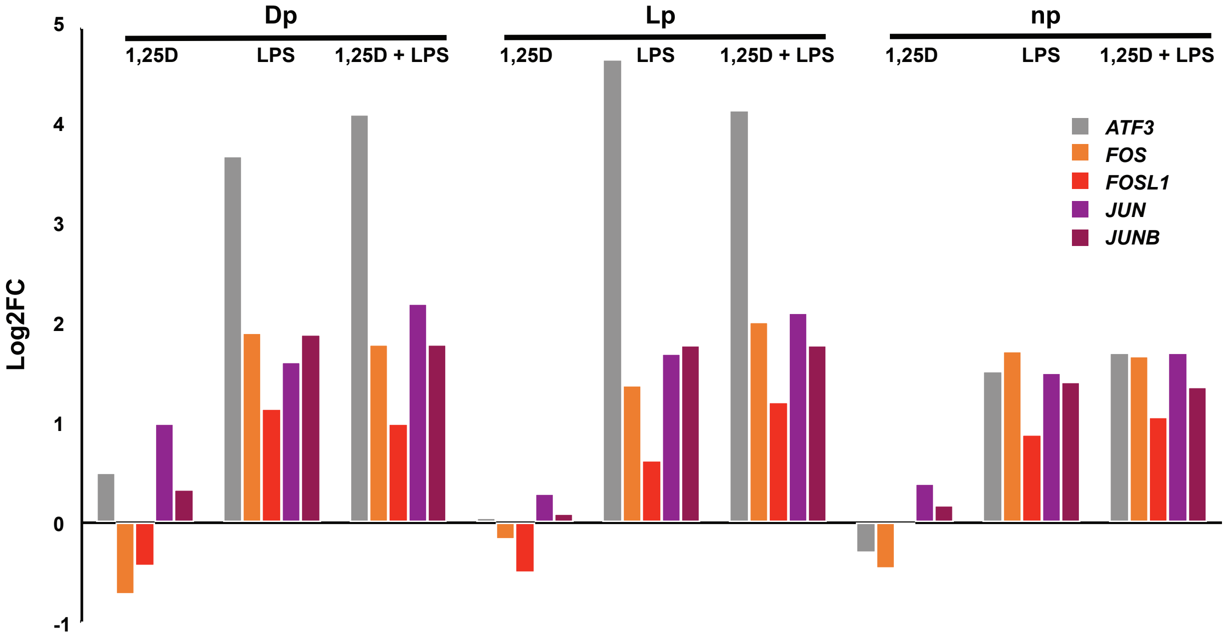
Dp
Lp
np
1,25D
LPS
1,25D + LPS
1,25D
LPS
1,25D + LPS
1,25D
LPS
1,25D + LPS
Log2FC
5
4
3
2
1
0
-1
ATF3
FOS
FOSL1
JUN
JUNB
